# Supplementary material for: Breakthrough: a first-in-class virtual simulator for dose optimization of ACE inhibitors in translational cardiovascular medicine
Source: Sci Rep. 2023 Feb 26;13:3300. doi: 10.1038/s41598-023-30453-x (PMC9968717; doi:10.1038/s41598-023-30453-x)
Supplement: Supplementary file 1 — Supplementary Table 1. [file 41598_2023_30453_MOESM1_ESM.docx]

Supplementary Table 1: Description of QSP Model Parameters

Definition of all mathematical symbols used for the description of the systems pharmacology model of benazeprilat pharmacokinetics and pharmacodynamics.

Ang(1-7)_fr_ ≝ amount of angiotensin (1-7) in free circulation compartment

Ang(1-7)_ts_ ≝ amount of angiotensin (1-7) in tissue compartment

AngIII_pl_ ≝ amount of angiotensin III in plasma compartment

AngIII_rn_ ≝ amount of angiotensin III in renal compartment

AngIII_ts_  ≝ amount of angiotensin III in tissue compartment

AngIV_pl_ ≝ amount of angiotensin IV in plasma compartment

AngIV_rn_ ≝ amount of angiotensin IV in renal compartment

AngIV_ts_ ≝ amount of angiotensin IV in tissue compartment

δ_24hr_ ≝ scale of renin production variance due to chronobiology

ε_Ang(1-7)_ ≝ proportional measurement error of angiotensin (1-7)

ε_AngI_  ≝ proportional measurement error of angiotensin I (1-10)

ε_AngII_  ≝ proportional measurement error of angiotensin II (1-8)

ε_AngIII_  ≝ proportional measurement error of angiotensin III (2-8)

ε_AngIV_ ≝ proportional measurement error of angiotensin IV (3-8)

ε_benazeprilat_ ≝ proportional measurement error of benazeprilat

E_fr_  ≝ amount of ACE in free circulation compartment

E_ts_  ≝ amount of ACE-bound benazeprilat in tissue compartment

EI_fr_  ≝ amount of ACE-bound benazeprilat in free circulation compartment

EI_ts_  ≝ amount of ACE-bound benazeprilat in tissue compartment

ES_fr_  ≝ amount of ACE-bound angiotensin I (1-10) in free circulation compartment

ES_ts_  ≝ amount of ACE-bound angiotensin I (1-10) in free tissue compartment

F_bio_  ≝ effective bioavailability of benazepril

f_CT_(t) ≝ effect of chronobiology on the production rate of the angiotensin I (1-10)

I_1abs, Ifr_ ≝ fraction of benazepril absorbed from depot to free circulation compartment

I_1abs, Ipr_  ≝ fraction of benazepril absorbed from depot to pre-circulation compartment

I_fr_  ≝ amount of benazeprilat in free circulation compartment

I_ns_  ≝ amount of benazeprilat bound via non-specific interactions in blood compartment

I_pr_ ≝ amount of benazeprilat in pre-circulation compartment

I_ts_  ≝ amount of benazeprilat in tissue compartment

k_–3_  ≝ rate of ACE-benazeprilat association *in vivo*

k_–1_  ≝ rate of ACE-angiotensin I (1-10) association *in vivo*

k_1_  ≝ rate of ACE-angiotensin I (1-10) disassociation *in vivo*

k_2_  ≝ rate of production of angiotensin II (1-8) from ACE-angiotensin I (1-10) *in vivo*

k_3_  ≝ rate of ACE-benazeprilat disassociation *in vivo*

ka ≝ zero-order analog absorption rate of benazepril

ka_1_  ≝ first-order analog absorption rate of benazepril

k_Cl, Ang(1-7)_ ≝ rate of clearance of angiotensin (1-7) from free circulation compartment; defined as

Cl_Ang(1-7)_/V_fr_

k_Cl, I_  ≝ rate of clearance of benazeprilat from free circulation compartment; defined as Cl_I_/V_fr_

k_Cl, P_  ≝ rate of clearance of angiotensin II (1-8) from free circulation compartment; defined

as Cl_P/_V_fr_

k_Cl, S_  ≝ rate of clearance of angiotensin I (1-10) from free circulation compartment; defined

as Cl_S_/V_fr_

k_Cl, AngIII_  ≝ rate of clearance of angiotensin III (1-8) from free circulation compartment; defined

as Cl_AngIII_/V_fr_

k_Cl, AngIV_  ≝ rate of clearance of angiotensin I (1-10) from free circulation compartment; defined

as Cl_AngIV_/V_fr_

k_fr, ns_  ≝ rate of transfer from free circulation compartment to non-specifically bound

interaction compartment; defined as Q_fr, ns_/V_fr_

k_fr, ts_  ≝ rate of transfer from free circulation compartment to tissue compartment defined as

Q_fr, ts_/V_fr_

k_I, 1-7_  ≝ overall in vivo conversion rate of angiotensin I (1-10) to angiotensin (1-7)

k_II, 1-7_  ≝ overall in vivo conversion rate of angiotensin II (1-8) to angiotensin (1-7)

k_II, III_  ≝ overall in vivo conversion rate of angiotensin II (1-8) to angiotensin III (2-8)

k_III, IV_  ≝ overall in vivo conversion rate of angiotensin III (2-8) to angiotensin IV (3-8)

k_ns, fr_  ≝ rate of transfer from non-specifically bound interaction compartment to free

circulation compartment; defined as Q_fr, ns_/V_ns_

k_pl, rn_  ≝ rate of transfer from plasma compartment to renal compartment, both within free

circulation compartment; defined as Q_rn_/V_pl_

k_pl, ts_  ≝ rate of transfer from plasma compartment within free circulation compartment to

tissue compartment; defined as k_fr, ts ∙_ (V_pl_/V_fr_)

k_rn, pl_  ≝ rate of transfer from renal compartment to plasma compartment, both within free

circulation compartment; defined as Q_rn_/V_rn_

k_rn, ts_  ≝ rate of transfer from renal compartment within free circulation compartment to

tissue compartment; defined as k_fr, ts ∙_ (V_rn_/V_fr_)

k_ts, fr_  ≝ rate of transfer from tissue compartment to free circulation compartment; defined as

Q_fr, ts_/V_ts_

k_ts, pl_  ≝ rate of transfer from tissue compartment to plasma compartment within free

circulation compartment; defined as k_ts, fr ∙_ (V_pl_/V_fr_)

k_ts, rn_  ≝ rate of transfer from tissue compartment to renal compartment within free

circulation compartment; defined as k_ts, fr ∙_ (V_rn_/V_fr_)

P_fr_ ≝ amount of angiotensin II (1-8) in free circulation compartment

PRA ≝ plasma renin activity

P_ts_ ≝ amount of angiotensin II (1-8) in tissue compartment

r_S_  ≝ production rate of angiotensin I (1-10)

S_fr_ ≝ amount of angiotensin I (1-10) in free circulation compartment

S_ts_  ≝ amount of angiotensin I (1-10) in tissue compartment

V_fr_  ≝ volume of distribution associated with free circulation compartment; defined as V_pl_ +

V_rn_

V_pl_  ≝ volume of distribution associated with plasma compartment within free circulation

compartment

V_rn_  ≝ volume of distribution associated with renal compartment within free circulation

compartment

V_ts_  ≝ volume of distribution associated with tissue compartment

Y_Ang(1-7)_  ≝ predicted concentration of angiotensin (1-7) in free circulation compartment

Y_AngI_  ≝ predicted concentration of angiotensin I (1-10) in free circulation compartment

Y_AngII_  ≝ predicted concentration of angiotensin II (1-8) in free circulation compartment

Y_AngIII_  ≝ predicted concentration of angiotensin III in free circulation compartment

Y_AngIV_  ≝ predicted concentration of angiotensin IV in free circulation compartment

Y_benazeprilat_ ≝ predicted concentration of benazeprilat in free circulation compartment
